# Impact of salt taste dysfunction on interdialytic weight gain for hemodialysis patients; a cross-sectional study

**DOI:** 10.1186/s12882-019-1312-3

**Published:** 2019-04-05

**Authors:** Mai Tanaka, Hiroki Nishiwaki, Hiroshi Kado, Yohei Doi, Chieko Ihoriya, Kenji Omae, Keiichi Tamagaki

**Affiliations:** 1 Department of Nephrology, Kyoto Prefectural University of Medicine, 465 Kajii-cho, Kamigyo-ku, Kyoto, 602-8566 Japan; 2 Department of Innovative Research and Education for Clinicians and Trainees, Fukushima Medical University, Fukushima, Japan; 3 Division of Nephrology, Department of Medicine, Showa University Fujigaoka Hospital, Yokohama, Kanagawa Japan; 4 Department of Nephrology, Omihachiman Community Medical Center, Omihachiman, Shiga Japan; 5 Department of Nephrology, Osaka Red Cross Hospital, Osaka, Japan; 6 Department of General Medicine, Kawasaki Medical School, Kurashiki, Okayama Japan

**Keywords:** Hemodialysis, Salt taste dysfunction, Interdialytic weight gain

## Abstract

**Background:**

Little is known about salt taste dysfunction among hemodialysis (HD) patients. This study aimed to elucidate the prevalence of salt taste dysfunction and its relationship with interdialytic weight gain (IDWG) among HD patients.

**Methods:**

A single-center cross-sectional study involving 99 maintenance HD patients was conducted in September 2015. Salt taste threshold was measured using a salt-impregnated test strip. Salt taste dysfunction was defined as a recognition threshold of ≥0.8%. IDWG was calculated as the mean value of weight gain at the beginning of each week during a 1-month period before the taste test. We performed a multivariate analysis using the standard linear regression model to investigate the association between salt taste dysfunction and IDWG.

**Results:**

Among the 99 participants, 42% had a recognition threshold of 0.6%, whereas 38% had a recognition threshold of ≥1.6%. Overall, the prevalence of salt taste dysfunction was 58%. The mean (±SD) IDWG was 4.9% (±1.7%), and there was no significant difference in IDWG between the two groups with (4.9%) and without (4.8%) salt taste dysfunction (*P* = 0.90). A multivariate analysis indicated that salt taste dysfunction is not significantly associated with IDWG (mean difference = 0.06; 95% confidence interval = − 0.27 to 0.40).

**Conclusions:**

The prevalence of salt taste dysfunction was very high among HD patients who had a unique distribution of salt taste recognition thresholds with two peaks. We found no significant association between salt taste dysfunction and IDWG.

## Background

Patients with chronic kidney disease (CKD), including those on hemodialysis (HD), commonly experience taste dysfunction for reasons such as zinc deficiency, dry mouth due to water loss, peripheral nerve disorders due to diabetes, adverse effects of medication, and uremia [1–6]. It was reported that as the kidney function worsens, taste dysfunction also worsens [7]. A previous study demonstrated that salt taste dysfunction was common in non-dialysis CKD patients, and that there was a positive correlation between salt taste dysfunction and salt intake [8]. For HD patients, high salt intake could lead to increased interdialytic weight gain (IDWG) [9, 10], which is a well-known prognostic factor for cardiovascular events and all-cause death [11, 12]. Moreover, it has been reported that many HD patients consume more salt than is recommended despite strong dietary salt restrictions [13]. A possible factor related to the disturbance of salt restriction is salt taste dysfunction, which makes the patient unable to sense the salt taste accurately, thus leading to consuming more salt than recommended.

Salt taste dysfunction could be influenced by a variety of factors, including age, sex, smoking, diabetes, and zinc levels [14–17]. Previous studies have demonstrated that excessive salt intake increased the salt taste function threshold [18] and that the administration of zinc [19] and a 1-week in-hospital CKD education program restricting dietary salt [8] improved salt taste dysfunction. However, to date, little is known about salt taste dysfunction among HD patients and its relationship with IDWG. Therefore, we hypothesized that the prevalence of salt taste dysfunction is higher among HD patients and that salt taste dysfunction has a significant role in increasing their IDWG through higher salt intake followed by increased fluid intake, as observed in general and non-dialysis CKD populations [8]. We conducted a cross-sectional study involving patients undergoing maintenance HD to elucidate the prevalence of salt taste dysfunction and the relationship between salt taste dysfunction and IDWG.

## Methods

### Participants

For this cross-sectional study, we recruited patients undergoing maintenance HD at Omihachiman Community Medical Center in September 2015. The conditions for participation were as follows: age 20 years or older; consistent outpatient HD three times per week; dialysis history of at least 6 months (duration of each dialysis session was not investigated); and consented to participate in this study.

The exclusion criteria were as follows: reduced cognitive function or inability to independently grant consent to participate in this study; psychiatric disorders; intraoral lesions (oral *Candida*, cancer of the tongue); active cancer (patients who underwent systematic cancer therapy within the past 5 years or who were scheduled to undergo therapy); active infection (patients with an infectious disease requiring systemic administration of an antibacterial or antifungal agent); inability to cooperate during the taste acuity test; missing body weight data for the month prior to the taste test; changes in dry weight (DW) during the month prior to the taste acuity test; and urinary output ≥1000 mL per day.

This study was conducted in accordance with the ethical guidelines of the Declaration of Helsinki and was approved by the Institutional Review Boards of Omihachiman Community Medical Center and Kyoto Prefectural University of Medicine (approval no. ERB-C-388). Written informed consents were obtained from all patients.

### Baseline demographics and clinical characteristics

We obtained the baseline data regarding the following potential confounding factors from the medical records: sex; age; BMI; HD vintage; causes of end-stage renal disease (diabetic nephropathy, glomerulonephritis, hypertensive nephrosclerosis, polycystic kidney disease, or others); comorbidities (diabetes, stroke, myocardial infarction, amputation, and femoral neck fracture); medications (renin-angiotensin system [RAS] inhibitor, diuretics, calcium channel blocker, β-blocker, statin, vitamin D, proton pump inhibitor, H_2_ blocker, sleep inducer, Chinese herbal medicine, and nonsteroidal anti-inflammatory drug); and laboratory data investigated immediately before the study. Data regarding history and current status of smoking, drinking, denture use, and education were obtained using questionnaires that patients completed at the time of the taste acuity test.

### Gustatory threshold for salt taste measurement

Salt taste dysfunction measurements were performed using a test strip to determine the threshold of salt taste acuity (SALSAVE salt-impregnated test strip; Advantec Toyo Co., Ltd., Tokyo, Japan) [20]. Patients placed seven SALSAVE test strips with different salt concentrations (0, 0.6, 0.8, 1.0, 1.2, 1.4, 1.6%) on their tongues in the order of lowest salt content to highest salt content. They were asked whether they tasted anything; if they replied affirmatively, then they were asked about what they tasted. The total time required for this simple test was approximately 3 min. The salt taste threshold measurable by SALSAVE was defined as the detection threshold for the lowest concentration that could be sensed when the participants tasted the strip and as the recognition threshold for the concentration that could be perceived as the taste of salt [20]. A previous study revealed that most of the healthy individuals had a recognition threshold of 0.6% [8]. Therefore, in this study, the individuals who could recognize the lowest concentration of 0.6% were considered to have normal salt taste acuity. If perception was impossible when the concentration reached 0.8% or more, then the individual was considered to have salt taste dysfunction. When the individual was unable to perceive any taste at all at the maximum detection threshold of 1.6%, the individual was considered to have ageusia. If the individual was able to perceive a taste but was unable to identify it as the taste of salt and instead identified it as another taste when the detection threshold was at the maximum of 1.4% or lower, then the individual was considered to have heterogeusia.

### Outcome measures

Body weight was measured (to the first decimal place) with an electronic calibrated scale before and after each dialysis session. IDWG, the primary outcome of this study, was calculated as the mean value of the rate of change in body weight (%) at the beginning of each week during a 1-month period before taste acuity test using the following formula:

$$ {\displaystyle \begin{array}{l}\left\{\left(\mathrm{pre}\hbox{-} \mathrm{dialysis}\ \mathrm{weight}\right)\hbox{-} \left(\mathrm{prior}\ \mathrm{post}\hbox{-} \mathrm{dialysis}\ \mathrm{weight}\right)\right\}\kern0.5em \\ {}\div \left(\mathrm{prior}\ \mathrm{post}\hbox{-} \mathrm{dialysis}\ \mathrm{weight}\right)\times 100\left(\%\right).\end{array}} $$

### Statistical analysis

Continuous variables were presented using the median value (interquartile range [IQR]). The nominal variables (categorical data) are presented as the number of participants and percentage.

Patients were divided into the two groups with or without salt taste dysfunction. We compared IDWG between the two groups using the Student’s t test. Assuming the prevalence of salt taste dysfunction in HD patients and standard deviation in IDWG as 75 and 1.5%, respectively, to detect a 1% absolute increase in IDWG from 5% in participants without salt taste dysfunction to 6% in those with salt taste dysfunction, with a two-sided significance level of 0.05, a total of 97 participants were required to have a power of 80%. We also explored the factors associated with salt taste dysfunction, including age, sex, HD vintage, smoking, dentures, diabetes, use of RAS inhibitors, and zinc level, by estimating odds ratios (ORs) and 95% confidence intervals (CIs) for the likelihood of having salt taste dysfunction (defined as a recognition threshold of ≥0.8%) using a multivariable logistic regression model. We further analyzed the association between salt taste dysfunction and IDWG by performing a multivariate analysis using the standard linear regression model. We adjusted for the following clinically important confounding factors related to both salt taste dysfunction and IDWG: age, sex, body mass index (BMI), HD vintage, diabetes, and serum albumin level. As a sensitivity analysis, we analyzed the association between salt taste dysfunction and IDWG calculated as the absolute change (not the change rate) using the same model as previously mentioned. Statistical analyses were performed using JMP11 (SAS Institute, Cary, NC, USA). Statistical significance was set at *P* < 0.05.

## Results

### Baseline characteristics

A total of 107 patients were enrolled in this study. After excluding five patients with missing body weight data, two patients with urinary output ≥1000 mL per day, and one patient with changes in DW during the study period, 99 patients were included in the analysis. Patient characteristics are shown in Table 1. Sixty-seven percent of patients were male. Their median age, HD vintage, and BMI were 67 (IQR, 60–74) years, 11 (IQR, 6–19) years, and 21.6 (IQR, 18.8–23.7) kg/m^2^, respectively. The most common cause of end-stage renal disease was glomerulonephritis (47%). One-half of the patients had dentures and 32% had diabetes. Fifty-nine percent of patients were current or past smokers, and 24% were using a RAS inhibitor. The median serum level of zinc was 52 (IQR, 46–59) μg/dL.

**Table 1:** Baseline characteristics of study participants

Variables	Total, *n* = 99
Male, n (%)	66 (66.7)
Median age, years (interquartile range)	67 (60–74)
Median BMI, kg/m^2^ (interquartile range)	21.6 (18.8–23.7)
Median HD vintage, years (interquartile range)	11 (6–19)
Median systolic blood pressure, mmHg (interquartile range)	137 (120–158)
Median diastolic blood pressure, mmHg (interquartile range)	72 (66–83)
Primary cause of end-stage renal disease, n (%)	
DMN	24 (24.2)
GN	46 (46.5)
HN	8 (8.1)
PKD	6 (6.1)
Others	15 (15.2)
Smoking (past, current)	58 (58.6)
Alcohol	19 (19.2)
Denture	50 (50.5)
More education than high school	67 (67.7)
More than 30 min of exercise, days per week	4 (0–7)
Comorbidities, n (%)	
Diabetes	32 (32.3)
Stroke	7 (7.1)
Myocardial infarction	15 (15.2)
Amputation	2 (2.0)
Femoral neck fracture	5 (5.2)
Laboratory data	
Median BUN, mg/dL (interquartile range)	54.7 (47.7–64.8)
Median creatinine, mg/dL (interquartile range)	11.1 (9.3–12.8)
Median hemoglobin, g/dL (interquartile range)	11.2 (10.6–11.8)
Median sodium, mEq/L (interquartile range)	139 (137–141)
Median phosphorus, mg/dL (interquartile range)	4.8 (4.2–5.3)
Median phosphorus, mg/dL (interquartile range)	4.8 (4.1–5.8)
Median zinc, μg/dL (interquartile range)	52 (46–59)
Median calcium, mg/dL (interquartile range)	8.6 (7.8–9.1)
Median albumin, g/dL (interquartile range)	3.4 (3.2–3.7)
Median β2 microglobulin, mg/L (interquartile range)	28.4 (25.6–31.5)
Medication, n (%)	
RAS inhibitor	24 (24.2)
Diuretics	13 (13.1)
Calcium channel blocker	47 (47.5)
Β-blocker	22 (22.2)
Statin	19 (19.2)
Vitamin D	36 (36.4)
Proton pump inhibitor	46 (46.5)
H_2_ blocker	9 (9.1)
Sleep inducer	13 (13.1)
Chinese herbal medicine	48 (48.5)
NSAIDs	4 (4.0)

Data are presented as number (percentage) or median (interquartile range). *BMI* body mass index, *HD* hemodialysis, *DMN* diabetic nephropathy, *GN* glomerulonephritis, *HN* hypertensive nephrosclerosis, *PKD* polycystic kidney disease, *RAS* renin-angiotensin system, *BUN* blood urea nitrogen, *NSAIDs* nonsteroidal anti-inflammatory drugs

### Salt taste threshold for hemodialysis patients

**Fig. 1:**
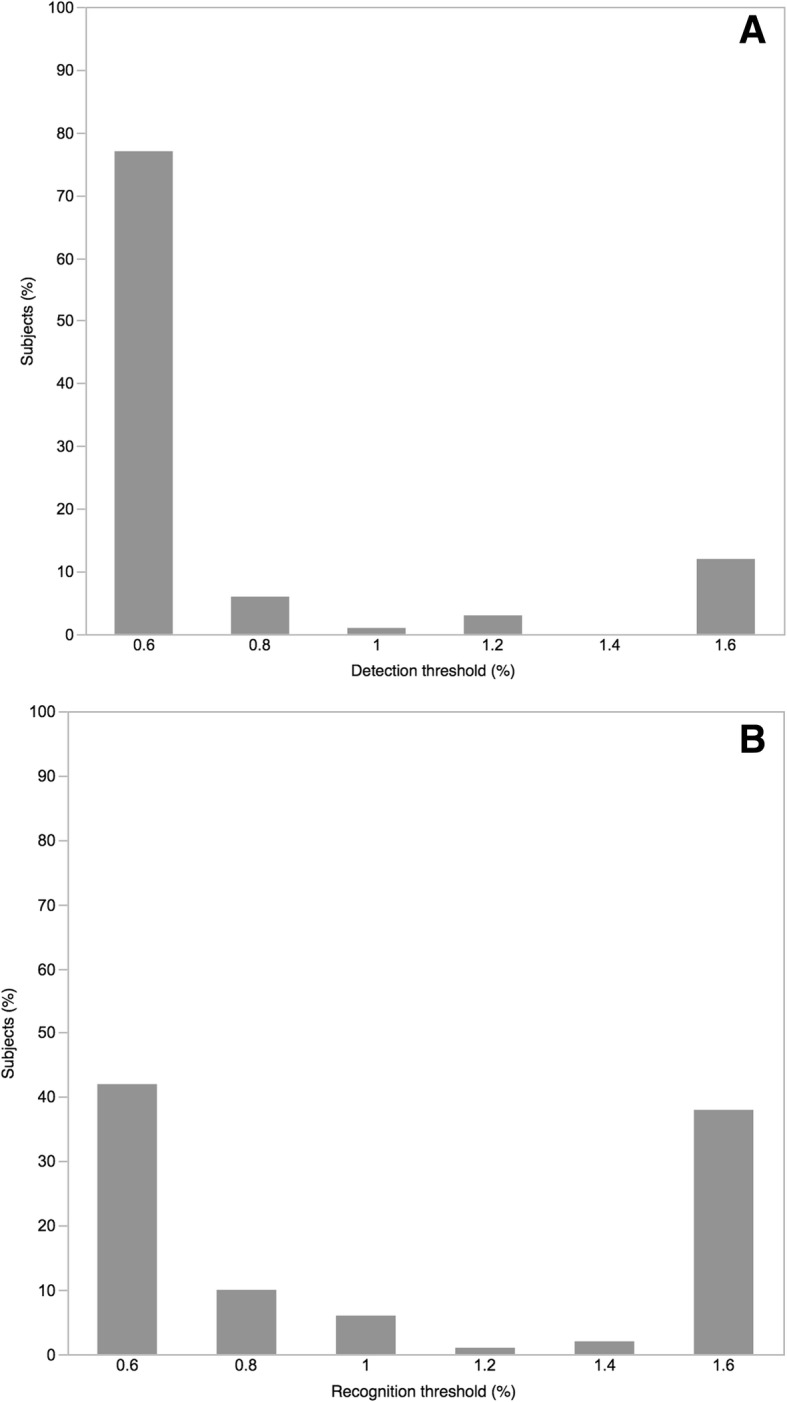
Distribution of salt taste threshold among study patients. **a** Distribution of detection threshold. Of all the participants, 78% had a detection threshold of 0.6%, whereas 12% had a threshold ≥1.6%. **b** Distribution of the recognition threshold. Data showed a bimodal distribution with peaks at 0.6% and ≥ 1.6% (42 and 38% of participants, respectively)

The salt taste threshold distribution is shown in Fig. 1. Seventy-eight percent of participants had a detection threshold of 0.6%, whereas 12% had a detection threshold of ≥1.6% (ageusia) (Fig. 1a). Recognition threshold data showed a bimodal distribution with peaks at 0.6% (normal) and ≥ 1.6% (42 and 38% of participants, respectively) (Fig. 1b). Fifty-eight percent of participants had salt taste dysfunction (i.e., recognition threshold of ≥0.8%), and 26% had heterogeusia. Moreover, the multivariate analysis revealed that male sex was significantly associated with salt taste dysfunction (OR vs female sex = 3.65; 95% CI = 1.10–13.3) (Table 2).

**Table 2:** Multivariable logistic regression analysis of factors related to salt taste dysfunction

Variables	Odds ratio (95% CI)	P
**Male vs female**	**3.65 (1.10–13.3)**	**0.03**
Age, per year	1.01 (0.97–1.05)	0.69
HD vintage, per year	0.97 (0.92–1.02)	0.30
Smoking, yes (vs no)	1.41 (0.41–4.55)	0.57
Dentures, yes (vs no)	1.43 (0.56–3.69)	0.45
Diabetes, yes (vs no)	0.46 (0.16–1.29)	0.14
RAS inhibitor, yes (vs no)	0.67 (0.24–1.87)	0.45
Zinc, per 1 μg/dL	0.99 (0.95–1.03)	0.72

*CI* confidence interval, *HD* hemodialysis, *RAS* renin-angiotensin system

*P* < 0.05 shown in bold

### Association between salt taste dysfunction and IDWG

**Fig. 2:**
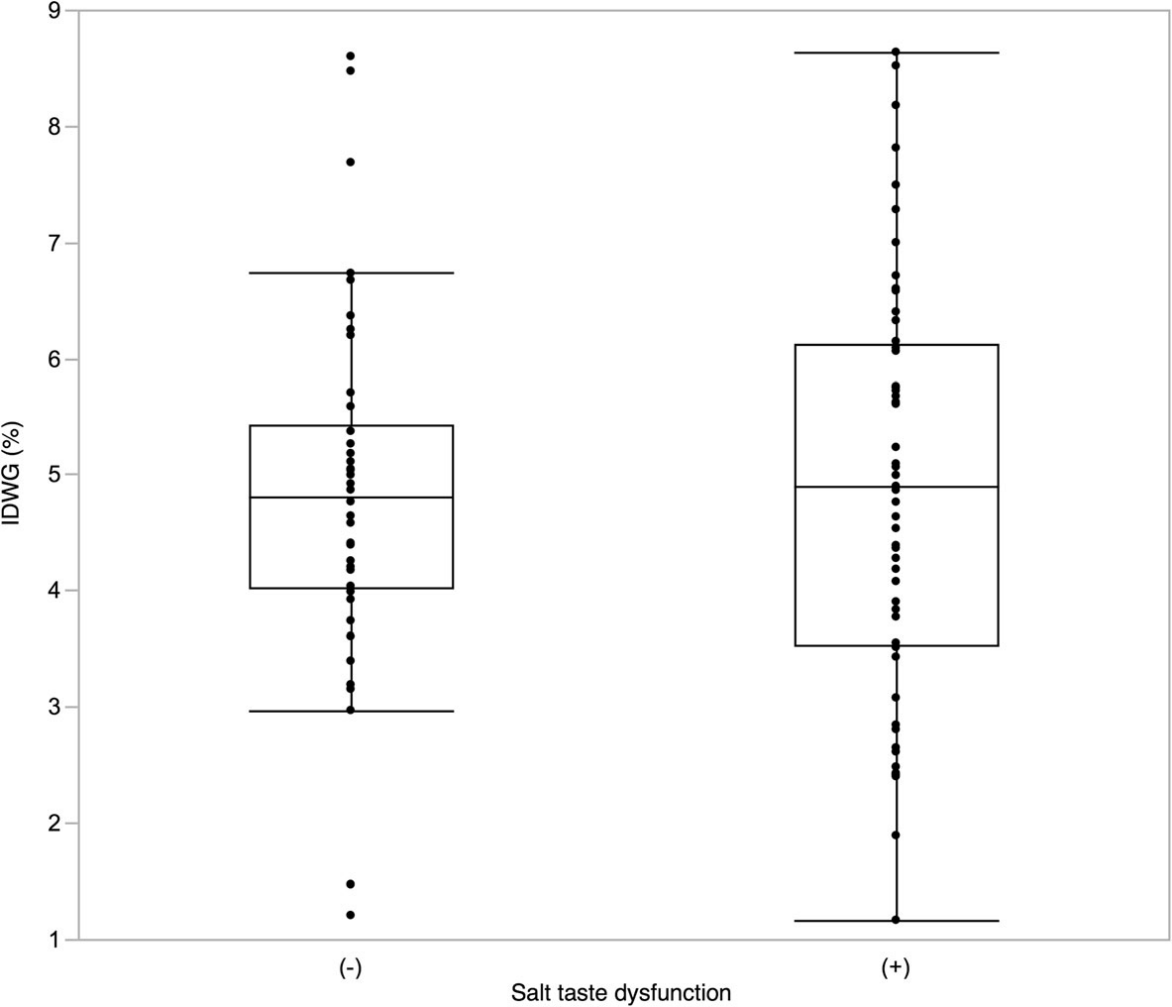
Difference between the presence or absence of salt taste dysfunction and interdialytic weight gain (IDWG). Fifty-eight percent of participants had salt taste dysfunction, which was defined as a recognition threshold ≥0.8%. There was no significant difference in IDWG between groups with and without salt taste dysfunction (*P* = 0.90)

The mean (±standard deviation [SD]) IDWG was 4.9% (±1.7%) for all participants. The mean IDWG values were 4.9 and 4.8% for groups with and without salt taste dysfunction, respectively, and there was no significant difference between the two groups (*P* = 0.90) (Fig. 2). The multivariate analysis indicated that there was no significant association between salt taste dysfunction and IDWG (mean difference = 0.06; 95% CI = − 0.27 to 0.40; *p* = 0.72) (Table 3). Sensitivity analysis confirmed that salt taste dysfunction was not associated with the absolute IDWG value (data not shown). However, HD vintage and BMI were negatively associated with IDWG.

**Table 3:** Multivariate linear regression analysis of factors related to interdialytic weight gain (%)

Variables	Mean difference (95% CI)	P
Salt taste dysfunction	0.06 (−0.27 to 0.40)	0.72
Male	−0.23 (−0.61 to 0.15)	0.23
**Age, per 1 year**	**− 0.04 (− 0.08 to 0.01)**	**0.01**
**HD vintage, per 1 year**	**−0.06 (− 0.10 to 0.02)**	**0.003**
**BMI, per 1 kg/m** ^**2**^	**−0.17 (− 0.27 to 0.06)**	**0.002**
Diabetes	0.06 (−0.32 to 0.44)	0.76
Albumin, per 1 g/dL	−0.17 (−1.19 to 0.84)	0.74

*CI* confidence interval, *HD* hemodialysis, *BMI* body mass index, *RAS* renin angiotensin system

*P* < 0.05 shown in bold

## Discussion

We conducted a semi-quantitative assessment of the salt taste threshold of HD patients. Interestingly, the salt taste recognition threshold of HD patients showed bimodal distribution peaks at 0.6% and ≥ 1.6%. Age, HD vintage, and BMI were independently associated with IDWG; however, we found no significant association between salt taste dysfunction and IDWG.

Intraoral dryness due to water loss, peripheral nerve disorders due to uremia and diabetes, medication-related adverse effects, and zinc deficiency are likely causes of CKD-related taste dysfunction; however, the mechanisms are not fully understood [1–6]. A recent study indicated that self-reported taste dysfunction was related to not only poor nutrition status but also all-cause mortality for HD patients [21].

The sensation of taste consists of five components: sweet, sour, salty, bitter, and savory. Among them, salt taste may have the most important role related to the consumption of salt by HD patients, which is associated with blood pressure, volume status, and prognosis [22]. For non-dialysis CKD patients, salt taste acuity was reported to be significantly correlated with urinary sodium excretion [22].

A previous study utilizing SALSAVE reported that 73% of healthy volunteers had a salt recognition threshold of 0.6%, whereas 71% of non-dialysis CKD patients had a salt recognition threshold of ≥0.8% (most commonly 0.8%). CKD patients had significantly higher salt recognition thresholds than healthy volunteers [8]. Therefore, we hypothesized that HD patients may have high salt recognition thresholds. As a result, interestingly, we found a unique distribution of the salt taste recognition threshold in HD patients, which was totally different from those previously observed in healthy individuals and non-HD CKD patients. However, exploration of the reasons for this unique distribution was beyond the scope of the present study; therefore, further studies are needed to investigate the mechanism of salt taste dysfunction.

In this study, we did not detect a significant relationship between salt taste dysfunction and IDWG. It has been generally believed that fluid intake is increased as more salt is ingested, which results in increased extracellular fluid volume [13]. One plausible explanation for this finding is that salt taste dysfunction affects the appetite differently among HD patients. Some may consume more salt due to salt taste dysfunction, but others may have appetite impairment. Furthermore, whether IDWG reflects salt intake is still debatable. McCausland et al. reported that salt intake during meals was positively correlated with IDWG, but this was not clinically significant [23]. They noted that the sodium burden of hypertonic dialysate, non-osmotic dry mouth due to post-dialysis hypotension, and habitual water-drinking may have more of an impact on IDWG than oral salt intake [23].

Our study had several strengths. First, to the best of our knowledge, this is the first study to quantitatively evaluate the association between salt taste dysfunction and IDWG in HD patients. Second, the SALSAVE test, which has been validated as a salt taste acuity test for both healthy individuals and CKD patients, is easy, inexpensive, and requires only a few minutes to perform [8]. Third, we evaluated IDWG 1 month before the taste acuity test to avoid the effects of behavioral changes after the taste acuity test, that is, the Hawthorne effect. Fourth, we adjusted for the relevant confounding factors that could affect the association between salt taste dysfunction and IDWG.

This study also had several limitations. First, this study was conducted at a single institution and included only Japanese patients, which may make it difficult to generalize the results of this study. Second, although the SALSAVE test can easily evaluate the gustatory threshold for salt taste as a screening test, its sensitivity has been questioned and the availability of impregnated salt concentrations is limited; therefore, we could not evaluate the taste thresholds accurately, especially those lower than 0.6% and higher than 1.6%. Third, our analysis may have been statistically underpowered to detect significant differences or relationships. Finally, we could not attribute causality to the associations between any exposure and IDWG because of the cross-sectional design of our study. Although our hypothesis that salt taste dysfunction causes IDWG is possible and biologically plausible, reverse causality could also exist. Nevertheless, no evidence that supports such an association has been generated.

## Conclusions

More than 50% of HD patients had salt taste dysfunction, and many patients also had heterogeusia and ageusia. Our findings suggest that more attention should be focused on salt taste when managing HD patients; however, no association was found between salt taste dysfunction and IDWG. Because of the limitations of our study, external validations incorporating mechanistic studies, especially measuring salt intake, which was considered the major intermediate factor in our hypothesis, and larger sample sizes would be helpful. Further studies evaluating the effectiveness of interventions to improve salt taste dysfunction, such as the administration of zinc and in-hospital education programs focused on restricting dietary salt, would also be helpful for HD patients with salt taste dysfunction.

## Abbreviations

BMI: body mass index
CI: confidence interval
CKD: chronic kidney disease
DW: dry weight
HD: hemodialysis
IDWG: increased interdialytic weight gain
IQR: interquartile range
OR: odds ratio
RAS: renin-angiotensin system
SD: standard deviation
TBW: total body water

## References

[1] Burge JC, Park HS, Whitlock CP, Schemmel RA (1979). Taste acuity in patients undergoing long-term hemodialysis. Kidney Int.

[2] Middleton RA, Allman-Farinelli MA (1999). Taste sensitivity is altered in patients with chronic renal failure receiving continuous ambulatory peritoneal dialysis. J Nutr.

[3] Burge JC, Schemmel RA, Park HS, Greene JA (1984). Taste acuity and zinc status in chronic renal disease. J Am Diet Assoc.

[4] Vreman HJ, Venter C, Leegwater J, Oliver C, Weiner MW (1980). Taste, smell and zinc metabolism in patients with chronic renal failure. Nephron.

[5] Ralli G, Magliulo G, Persichetti S, Maggi S, Farina Mazzeo A (1985). Electrogustometry in hemodialysis patients. Acta Otorhinolaryngol Belg.

[6] Fernstrom A, Hylander B, Rossner S (1996). Taste acuity in patients with chronic renal failure. Clin Nephrol.

[7] Takeda S, Matsuzaka K, Kobayashi F, Nakashima A, Hara Y, Kimura Y (2012). Relation of estimated glomerular filtration rate (eGFR) to patients with taste abnormalities. Japan Soc Evidence Dental Prof.

[8] Kusaba T, Mori Y, Masami O, Hiroko N, Adachi T, Sugishita C (2009). Sodium restriction improves the gustatory threshold for salty taste in patients with chronic kidney disease. Kidney Int.

[9] Kayikcioglu M, Tumuklu M, Ozkahya M, Ozdogan O, Asci G, Duman S (2009). The benefit of salt restriction in the treatment of end-stage renal disease by haemodialysis. Nephrol Dial Transplant.

[10] Maduell F, Navarro V (2000). Dietary salt intake and blood pressure control in haemodialysis patients. Nephrol Dial Transplant.

[11] Kalantar-Zadeh K, Regidor DL, Kovesdy CP, Van Wyck D, Bunnapradist S, Horwich TB (2009). Fluid retention is associated with cardiovascular mortality in patients undergoing long-term hemodialysis. Circulation..

[12] Leggat JE, Orzol SM, Hulbert-Shearon TE, Golper TA, Jones CA, Held PJ (1998). Noncompliance in hemodialysis: predictors and survival analysis. Am J Kidney Dis.

[13] Wright JA, Cavanaugh KL (2010). Dietary sodium in chronic kidney disease: a comprehensive approach. Semin Dial.

[14] Schiffman SS (1997). Taste and smell losses in normal aging and disease. JAMA.

[15] Le Floch JP, Le Lievre G, Labroue M, Peynegre R, Perlemuter L (1992). Early detection of diabetic patients at risk of developing degenerative complications using electric gustometry: a five-year follow-up study. Eur J Med.

[16] Redington K (1984). Taste differences between cigarette smokers and nonsmokers. Pharmacol Biochem Behav.

[17] Mafra D, Cuppari L, Cozzolino SM (2002). Iron and zinc status of patients with chronic renal failure who are not on dialysis. J Ren Nutr.

[18] Huggins RL, Di Nicolantonio R, Morgan TO (1992). Preferred salt levels and salt taste acuity in human subjects after ingestion of untasted salt. Appetite.

[19] Mahajan SK, Prasad AS, Lambujon J, Abbasi AA, Briggs WA, McDonald FD (1980). Improvement of uremic hypogeusia by zinc: a double-blind study. Am J Clin Nutr.

[20] Nishimoto K, Hirota R, Egawa M, Furuta S (1996). Clinical evaluation of taste dysfunction using a salt-impregnated taste strip. ORL J Otorhinolaryngol Relat Spec.

[21] Lynch KE, Lynch R, Curhan GC, Brunelli SM (2013). Altered taste perception and nutritional status among hemodialysis patients. J Ren Nutr.

[22] Mc Causland FR, Waikar SS, Brunelli SM (2013). The relevance of dietary sodium in hemodialysis. Nephrol Dial Transplant.

[23] McCausland FR, Waikar SS, Brunelli SM (2012). Increased dietary sodium is independently associated with greater mortality among prevalent hemodialysis patients. Kidney Int.

